# Anthrax Edema Toxin Modulates PKA- and CREB-Dependent Signaling in Two Phases

**DOI:** 10.1371/journal.pone.0003564

**Published:** 2008-10-29

**Authors:** Andrea Puhar, Federica Dal Molin, Stéphanie Horvath, Daniel Ladants, Cesare Montecucco

**Affiliations:** 1 Dipartimento di Scienze Biomediche Sperimentali, Università di Padova, Padova, Italy; 2 Unité de Biochimie des Interactions Macromoléculaires, CNRS URA 2185, Institut Pasteur, Paris, France; Columbia University, United States of America

## Abstract

**Background:**

Anthrax edema toxin (EdTx) is an adenylate cyclase which operates in the perinuclear region of host cells. However, the action of EdTx is poorly understood, especially at molecular level. The ability of EdTx to modulate cAMP-dependent signaling was studied in Jurkat T cells and was compared with that of other cAMP-rising agents: *Bordetella pertussis* adenylate cyclase toxin, cholera toxin and forskolin.

**Methodology/Principal Findings:**

EdTx caused a prolonged increase of the intracellular cAMP concentration. This led to nuclear translocation of the cAMP-dependent protein kinase (PKA) catalytic subunit, phosphorylation of cAMP response element binding protein (CREB) and expression of a reporter gene under control of the cAMP response element. Neither p90 ribosomal S6 kinase nor mitogen- and stress-activated kinase, which mediate CREB phosphorylation during T cell activation, were involved. The duration of phospho-CREB binding to chromatin correlated with the spatio-temporal rise of cAMP levels. Strikingly, EdTx pre-treated T cells were unresponsive to other stimuli involving CREB phosphorylation such as addition of forskolin or T cell receptor cross-linking.

**Conclusions/Significance:**

We concluded that, in a first intoxication phase, EdTx induces PKA-dependent signaling, which culminates in CREB phosphorylation and activation of gene transcription. Subsequently CREB phosphorylation is impaired and therefore T cells are not able to respond to cues involving CREB. The present data functionally link the perinuclear localization of EdTx to its intoxication mechanism, indicating that this is a specific feature of its intoxication mechanism.

## Introduction

Anthrax is caused by *Bacillus anthracis*, a Gram-positive, sporulating bacterium. This organism is commonly found in soil and it mainly infects large herbivores, but occasionally also humans. The spores can enter the body from skin abrasions, the gastro-intestinal tract, or the lungs. Whilst cutaneous anthrax has only local effects, the gastro-intestinal and inhalation forms may lead to a systemic infection, causing septicemia and toxemia. Fatality of inhalation anthrax is very high [1], [2]. Even though *B. anthracis* is sensitive to different antibiotics, their therapeutic benefit is frequently diminished by the late onset of symptoms. Therefore, in recent years much research focused at finding new therapeutics that block the action of anthrax toxins, which are major virulence factors of *B. anthracis* [3].

Pathogenic strains of *B. anthracis* harbor three plasmid-encoded virulence factors: a polyglutamic capsule and two A–B toxins [2], [4]. These toxins consist of two enzymatic components, edema factor (EF) and lethal factor (LF) which share their B carrier, termed anthrax protective antigen (PA) [5]. PA can associate with two cell surface receptors, tumor endothelial marker 8 (TEM8) and capillary morphogenesis protein 2 (CMG2) [6], [7], and possibly with the co-receptor low-density lipoprotein receptor-related protein LRP6 [8]–[10]. On to the cell surface, PA forms a heptamer that binds up to three molecules of EF or LF [5]. After endocytosis, at low pH, the heptamer dissociates from the receptors and inserts into the lipid bilayer forming a pore through which partially unfolded EF and LF cross the membrane [11]. The slightly acidic pH of early endosomes is sufficient to mediate the detachment of toxins from TEM8, but the more acidic pH of late endosomes (LEs) is required for their dissociation from CMG2 [12]. However, it was proposed that LF rarely translocates directly to the cytosol from the limiting membrane of endosomes; more frequently it is delivered to intralumenal vesicles (ILVs) which then release the toxin upon back-fusion with the limiting membrane at the LE stage [13]–[15]. EF was found to remain attached to the cytosolic side of LE membrane, whereas LF freely diffuses into the cytosol [13], [16], [17].

EF and LF act on many cell types, but their action on cells of both innate and adaptive immunity appears particularly relevant as it allows *B. anthracis* to survive the host defense mechanisms. In some cell types, the two toxins act in synergism [18], [19]. EF and LF affect fundamental signaling pathways linking extracellular stimuli to cell function. LF is a Zn-dependent metalloprotease that cleaves the N-terminal portion of most isoforms of the mitogen activated protein kinase kinases (MAPKKs or MEKs) [20], thus disrupting MEK-dependent signaling [5], [19]. The action of EF is less understood. EF is a calmodulin-dependent adenylate cyclase that perturbs ion homeostasis and cell signaling by increasing the cytosolic cAMP concentration [5], [19]. Injection of PA+EF (edema toxin, EdTx) into mice causes tissue lesion and death [21].

EdTx-induced alterations of cell signaling are generally thought to be inhibitory and to be mediated by cAMP-dependent protein kinase (PKA) [19]. In particular, CD4^+^ T cells were identified as targets of anthrax toxins *in vitro* and *ex vivo* [22]–[24]. EdTx is able to suppress T cell proliferation and to inhibit cytokine release [22], [24]. MEK, ERK and JNK phosphorylation were found to be affected by EdTx in cells activated by T cell receptor (TCR) cross-linking, which resulted in reduced activity of the transcription factors NF-AT and AP-1 [22], [24]. Similarly, EdTx inhibits T cell chemotaxis by downregulation of ERK phosphorylation [25]. These effects were attributed to the inhibitory action of PKA on upstream kinases of the MAPK family, among which Raf. Nevertheless, targets of PKA in EdTx-triggered signaling are scarcely defined.

Using Jurkat T cells, we studied the impact of EdTx-mediated intracellular cAMP elevation on PKA- and cAMP response element binding protein (CREB)-dependent signaling and demonstrated that EdTx activates gene expression. We report that EdTx generates an extremely enduring increase in cAMP levels, which stimulates nuclear translocation of the PKA catalytic subunit and phosphorylation of CREB. This in turn allows binding of phosphorylated CREB (pCREB) to chromatin and transcription of a reporter gene under control of the cAMP response element (CRE). Further, we found that pre-treatment with EdTx prevents phosphorylation of CREB induced by a cAMP stimulus or by TCR cross-linking. Comparison with other cAMP-elevating agents showed that length and site of cAMP synthesis are crucial to modulation of gene transcription, which indicates that prolonged perinuclear production of cAMP is a specific and functional feature of EdTx intoxication mechanism.

## Materials and Methods

### Reagents, plasmids, and proteins

All chemicals were p.a. grade or higher and purchased from Sigma, together with forskolin, cholera toxin (CT), and poly-D-lysine. Protease inhibitor tablets (complete, devoid of EDTA) were purchased from Roche. Adefovir dipivoxil was a kind gift of Gilead (CA, USA). Roswell Park Memorial Institute Medium (RPMI) 1640 and fetal bovine serum (FBS) were bought from Invitrogen. Antibiotics and L-glutamine were purchased from GIBCO. Antibodies against CREB and pCREB (phospho-S133) were purchased from Upstate, against tetra-acetyl-histone H4, pRSK1/2 p90 (phospho-S380), pMSK1 (phospho-T581) from Abcam, against CD3 (OKT3) from eBioscience, against actin from Sigma. Secondary antibodies and H-89 dihydrochloride were obtained from Calbiochem. Plasmids pcDNA3-RII-CFP and pcDNA3-C-YFP coding, respectively, for the regulatory subunit of PKA fused to cyan fluorescent protein and for the catalytic subunit of PKA fused to yellow fluorescent protein were as in [26]. Plasmids pCRE-Luc and pRL-TK were obtained from Clontech and Promega, respectively. PA [27], EF [16], and CyaA [28] were prepared as previously described. Endotoxin contamination was monitored with PyroGene-rFC Endotoxin Detection System (Lonza) and was found in all preparations to be below 0.5 endotoxin units/µg protein.

### Cell culture

E6.1 Jurkat T cells were kept in RPMI 1640, supplemented with 100 U/ml penicillin, 100 µg/ml streptomycin, 2 mM L-glutamine, 10 mM Hepes and 10% heat-inactivated FBS. Cells were maintained at 37°C and 5% CO_2_ in a humid environment.

### Cell viability test

Viability of Jurkat cells was assessed by measuring the oxidation of 3-(4,5-dimethylthiazol-2-yl)-5-(3-carboxymethoxyphenyl)-2-(4-sulfophenyl)-2H-tetrazolium) (MTS) using the CellTiter96 Aqueous One Solution Cell Proliferation Assay (Promega) 12 h, 24 h, 48 h, and 72 h after treatment with 10 nM EF+40 nM PA (final concentration hereafter), 3 nM CT, 5 nM CyaA, 25 µM forskolin (in DMSO, from a 10 mM stock solution), 1∶400 (vol/vol) DMSO, 10 nM EF, or 40 nM PA.

### Determination of the cellular content of cAMP

5×10^5^ Jurkat cells in 200 µl were prepared the evening before the determination and stimulated for the indicated times with 10 nM EF+40 nM PA, 3 nM CT, 5 nM CyaA, 25 µM forskolin, or left untreated. 5 µM adefovir dipivoxil (in DMSO, from a 0.5 mM stock solution) was added 2 to 12 h before stimulation with EdTx and was reapplied 4 h after the first addition. Samples were prepared in triplicates. The culture medium was removed after 10 min of centrifugation at 1300 rpm and 4°C and the cells immediately processed for intracellular cAMP measurement by enzyme-linked immunoassay following the manufacturer's instruction (Biotrak EIA, GE Healthcare). The number of cells present in each sample was determined separately using a Bürker chamber.

### Plasmid preparation and transfection of Jurkat cells

Plasmids pcDNA3-C-YFP, pcDNA3-RII-CFP, pCRE-Luc, and pRL-TK were propagated in CaCl_2_-competent *Escherichia coli* XL-1Blue cells that were transformed by the heat shock method [29]. To purify plasmid DNA, a Maxi-Prep (QIAGEN) was performed according to the manufacturer's instructions. 9×10^6^ of Jurkat cells in 30 ml of culture medium were prepared the evening before transfection. 20 µg each of pcDNA3-RII-CFP and pcDNA3-C-YFP or 20 µg pCRE-Luc and 1 µg pRL-TK were introduced into cells kept in 400 µl of culture medium devoid of FBS giving an electric shock at 250 V and 950 F in electroporation cuvettes with 0.4 cm gap (Bio-Rad) using a GenePulser Xcell electroporator (Bio-Rad). The FBS content was brought back to 10% and cells allowed to grow a few hours at a concentration of 5×10^5^ cells/ml.

### Imaging of the nuclear translocation of PKA catalytic subunit

48 h after transfection with pcDNA3-RII-CFP and pcDNA3-C-YFP, cells were stimulated with 10 nM EF+40 nM PA, 3 nM CT, 5 nM CyaA, 25 µM forskolin, or left untreated and at the indicated times allowed to adhere for 10 min to cover slips coated with poly-D-lysine (50 µg/ml). Cells were paraformaldehyde-fixed according to standard protocols. Z-stacks of samples with 0.27 µm width were acquired at 490 nm on a DMIRE2 fluorescence microscope (Leica), equipped with a Leica DC 500 CCD camera, using a 63× oil immersion objective with NA 1.4 and 1.5× magnification. Images were deconvoluted with the software LeicaDeblur and the maximal projection created in WCIF Image J [30].

### Western blotting

Jurkat cells were treated for the indicated times with 10 nM EF+40 nM PA, 3 nM CT, 5 nM CyaA, 25 µM forskolin, or left untreated. To induce TCR cross-linking, cells were transferred for 30 min to plates coated over night with antibody against CD3 (10 µg/ml in PBS). Samples containing 10^5^ cells were lysed on ice for 10 min under rotation in the presence of protease inhibitors and heated for 8 min to 95°C in 60 µl Laemmli buffer [31]. 10 µg of protein per lane were subjected to SDS-PAGE on 12% polyacrylamide gels at room temperature and 20 mA per gel and transferred at 200 mA to nitrocellulose in a refrigerated chamber. Membranes were incubated with the appropriate antibodies following the manufacturer's instructions. When necessary, membranes were stripped by 30 min incubation at 50°C in 62.5 mM Tris/HCl pH 6.7, 2% (w/v) SDS, 1 mM 2-mercaptoethanol, thoroughly rinsed and re-probed. Chemiluminescence was developed with ECL Plus or ECL Advance western blotting detection system (GE Healthcare) and emission was measured with ChemiDoc XRS (Bio-Rad). Band intensities were quantified on the original files with the software Quantity One (Bio-Rad). None of the bands was saturated. Band intensities of blots against CREB, pCREB, pRSK1/2 and pMSK1 were corrected with the intensities of the loading control actin and set equal to 1 at t = 0.

### Luminescence assay

The day after transfection with pCRE-Luc and pRL-TK, cells were stimulated with 10 nM EF+40 nM PA, 3 nM CT, 5 nM CyaA, or 25 µM forskolin. As controls, 1∶400 (vol/vol) DMSO, 10 nM EF, or 40 nM PA were added or cells were left untreated. Cells were lysed on ice for 10 min in the presence of protease inhibitors and luciferase activity was assayed immediately using the Dual-Luciferase Reporter Assay System (Promega) as described by the manufacturer. For each time point, 10^5^ cells were present. Samples were prepared in triplicates. For every sample, both the ratio between the luminescence of firefly and renilla luciferases and the firefly luciferase activity per µg of protein in cell lysates were obtained. Proteins were quantified by the Bradford method (Biorad Protein Assay).

### Chromatin immunoprecipitation

The evening before the experiment, 5×10^5^ Jurkat cells were inoculated into 2 ml culture medium. The following day, they were treated for the indicated times with 10 nM EF+40 nM PA, or 3 nM CT, or 5 nM CyaA, or 25 µM forskolin, or DMSO (1∶400 vol/vol), or left untreated. Proteins and DNA were cross-linked by addition of 1% (w/v) formaldehyde directly to the culture medium and incubation for 10 min at 37°C. The reaction was stopped with 125 mM glycine in PBS. Cells were washed with ice-cold PBS containing protease inhibitors and lysed on ice for 10 min under rotation in the presence of protease inhibitors. DNA was sheared to fragments of about 600 bp by three cycles of 10 s of ultrasonic dispersion using a Fisher Sonic Dismembrator Model 300 at 30% power output with a 4 mm tip. The lysates were subjected to chromatin immunoprecipitation according to the manufacturer instructions using the kit produced by Upstate, including standard controls such as absence of antibody and presence of unspecific rabbit IgG. 15 µl of anti-pCREB antibody were employed. Samples were processed for SDS-PAGE and subjected to western blotting with an antibody against tetra-acetyl-histone H4.

## Results

### Time course of cAMP production

The concentrations of the toxins and drugs used here were deduced from the literature. In fact, the concentration of EdTx, CT, and CyaA in circulating fluids during pathogenesis is not yet known. The selected toxin concentrations were either within or below the affinity range of these toxins for their respective cell surface receptor [32]–[36]. One can reasonably hypothesize that such toxin concentrations will be found *in vivo*. In particular, the present concentration of 10 nM EdTx was chosen on the basis of our previous cellular studies [16], [24], [25] and is by far lower than the estimated 150 nM tissue fluid concentration used in a recent study of EdTx toxicity in mice [21]. Indeed, cell viability and growth were unaffected by EdTx, CT, CyaA, and forskolin at the concentrations we employed for the duration of the experiments (not shown).

**Figure 1:**
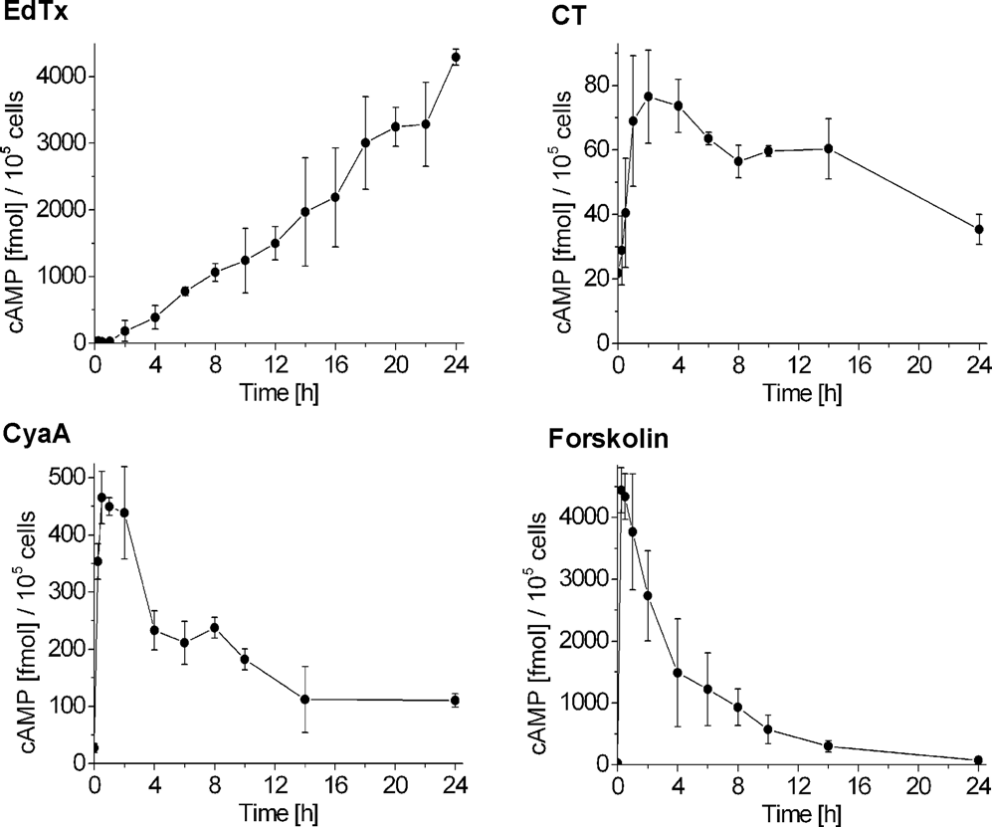
Time course of cAMP production by Jurkat cells stimulated with EdTx, CT, CyaA, or forskolin. Jurkat T cells were stimulated for the indicated times with EdTx (10 nM EF+40 nM PA), CT (3 nM), CyaA (5 nM), or forskolin (25 µM). At the end of the incubations, the samples, which contained 10^5^ cells, were lysed and immediately subjected to competition ELISA in order to determine the amount of cAMP present. Adefovir dipivoxil, a specific inhibitor of EdTx, abolished cAMP synthesis in EdTx-treated cells (not shown). Note the different cAMP scales in the four panels. Experiments were not normalized to percentage values to provide the actual levels of cAMP. Data are the mean and standard deviation of four independent experiments for EdTx and three for CT, CyaA and forskolin, performed in triplicates.

The time course of total intracellular cAMP synthesis in Jurkat T cells following treatment with EdTx, CT, CyaA and forskolin was determined by competition ELISA. Although this approach is not as sensitive as the previously used FRET-based live imaging of intracellular cAMP production [16], it allows to measure the total cAMP content over a prolonged period of time. All four agents induced a rise of intracellular cAMP levels, however the kinetics and the amounts of produced cAMP strongly differed among them (Fig. 1). Addition of EdTx (10 nM EF+40 nM PA) lead to a rise of the intracellular cAMP concentration that was appreciable after 2 h and, strikingly, increased steadily during the following 24 h, producing up to 4300 fmol of cAMP per 10^5^ Jurkat cells. Using FRET-based live imaging of EdTx activity, we previously found that cAMP levels start rising 30–40 min after toxin addition [16]. Synthesis of cAMP was suppressed by adefovir dipivoxil (not shown), a specific inhibitor of EdTx [37]. Stimulation of Jurkat cells with 3 nM CT induced a rapid, but limited rise in intracellular cAMP concentration that attained the highest level 2 h after toxin addition (about 80 fmol of cAMP per 10^5^ cells) and then slowly decayed. Addition of 5 nM CyaA induced an immediate rise in cAMP concentration that reached its maximum 30–60 min after addition with about 450 fmol per 10^5^ Jurkat cells, maintained these cAMP levels for another 1.5 h, and then progressively decayed. Forskolin (25 µM) caused a rapid (15 min) and sharp peak of cAMP concentration with 4400 fmol of cAMP per 10^5^ Jurkat cells.

### Activation of PKA

**Figure 2:**
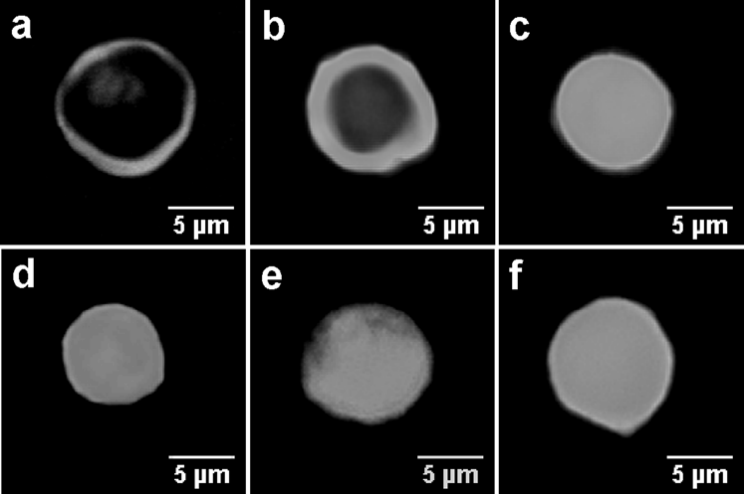
Activation and nuclear translocation of PKA catalytic subunit upon stimulation of Jurkat cells with EdTx, CT, CyaA, or forskolin. Cells were transiently transfected with a fluorescent probe coding for the PKA catalytic subunit fused to yellow fluorescent protein, stimulated with EdTx (10 nM EF+40 nM PA), CT (3 nM), CyaA (5 nM), or forskolin (25 µM), fixed and observed at the microscope. a) in resting cells, the catalytic subunit of PKA resides within the ring-shaped cytoplasm. b) 1.5 h after addition of EdTx, the majority of the PKA catalytic subunit is still present in the cytosol, whilst c) after 8 h it is inside the nucleus. The migration of PKA catalytic subunit induced by CT, CyaA, and forskolin after 1.5 h are shown in panels d), e) and f), respectively.

Next, we tested the possibility that the toxin- or forskolin-induced rise in cAMP concentration could activate PKA and drive the consequent nuclear translocation of its catalytic subunit. We imaged PKA in cells by transiently expressing a fluorescent probe consisting of the PKA catalytic subunit fused to YFP. As expected, in resting Jurkat cells the catalytic subunit of PKA resided in the ring-shaped cytosol (Fig. 2a). To the contrary, upon stimulation with EdTx (10 nM EF+40 nM PA), CT (3 nM), CyaA (5 nM), or forskolin (25 µM) the catalytic subunit translocated to the nucleus (Fig. 2b–f). According to the respective kinetics of intracellular cAMP increase of the different toxins, the catalytic subunit of PKA diffused into the nucleus with different time courses.

### Phosphorylation of CREB

**Figure 3:**
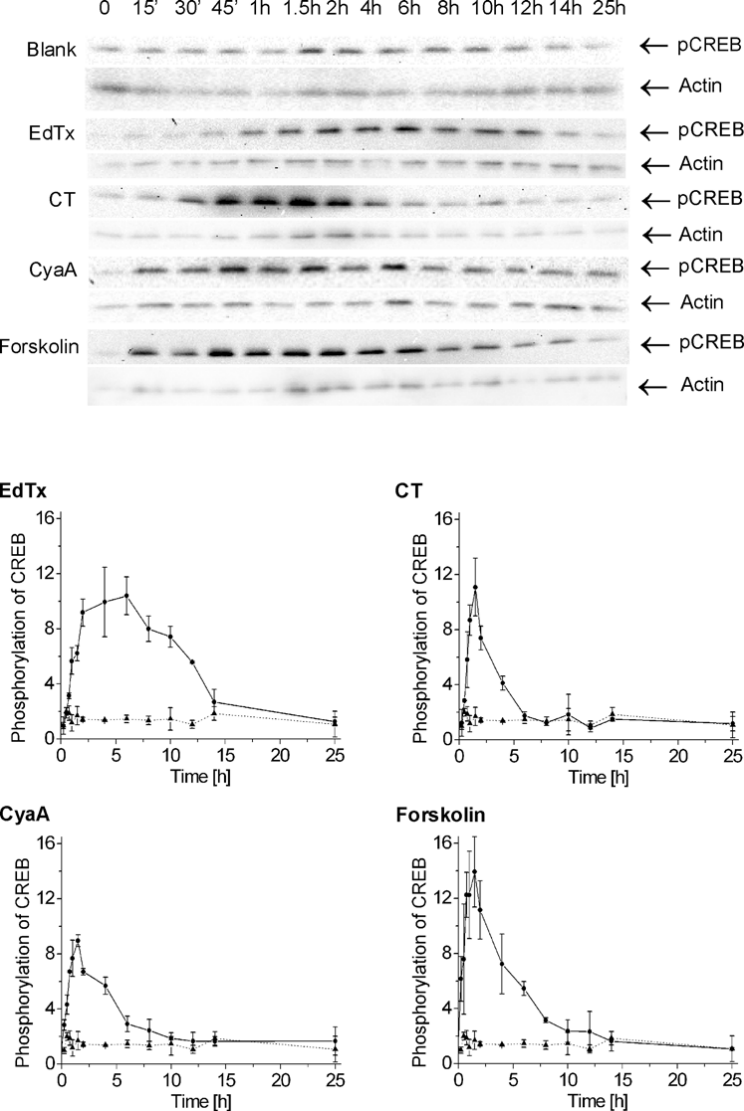
Phosphorylation of CREB upon stimulation of Jurkat cells with EdTx, CT, CyaA, or forskolin. Cells were stimulated for the indicated times, lysed and processed for western blotting using an antibody against S133-pCREB. On each lane 10 µg of protein were loaded. Upper panel, western blots depicting the time course of EdTx (10 nM EF+40 nM PA), CT (3 nM), CyaA (5 nM), and forskolin (25 µM) dependent CREB phosphorylation. Lower panels, the graphs report the quantitative analysis of the kinetics of CREB phosphorylation induced by EdTx, CT, CyaA, and forskolin (solid line with filled circles). Untreated samples are depicted by the dotted line with black triangles. The intensity of each band was determined using the software Quantity One (Bio-Rad) and was corrected for different loadings using the corresponding value of actin as control. The value at time 0 was set equal to 1. The total amount of cellular CREB did not change during the time course of CREB phosphorylation (not shown). The blots show one representative experiment and the data in the graphs are the mean and standard deviation of three independent experiments.

This result suggested to study the functional consequence of the nuclear migration of PKA on the phosphorylation of CREB, which is the best characterized cAMP-regulated transcription factor [38]–[41]. Western blot analysis of the active, S133-phosphorylated form of CREB from EdTx (10 nM EF+40 nM PA), CT (3 nM), CyaA (5 nM), or forskolin (25 µM) stimulated cells are shown in Fig. 3. A precise quantification of CREB phosphorylation kinetics was then obtained from the ratios of the pCREB signal to that of the actin level, which were determined on the same blots with an actin-specific antibody (not shown). The time course of CREB phosphorylation resembled the time course of cAMP production, except for EdTx at late time points. Indeed, EdTx action started with a delay of about 45 min and then exhibited a strongly sustained CREB phosphorylation, which reached the maximum around 6 h (Fig. 3). Notably, a high level of pCREB was maintained until 12 h after toxin addition, but it then returned to basal levels in spite of the rising concentration of intracellular cAMP (Fig. 1), most likely owing to the activation of negative feedback mechanisms [41]. CT had a lag of about 45 min before significant CREB phosphorylation could be observed, which peaked 1.5 h after stimulation and came down to basal levels after 6 h (Fig. 3). At the maxima, the amounts of pCREB produced in response to CT were comparable to those produced by EdTx. CyaA triggered a very rapid phosphorylation of CREB (appreciable 15 min after toxin addition), reaching a maximum after 1.5 h and followed by a smooth decay that was completed after 10–12 h (Fig. 3). At their maxima, the quantity of pCREB produced by CyaA was comparable to those induced by EdTx and CT. Finally, forskolin mediated a time course of CREB phosphorylation that was very similar to that of CyaA (Fig. 3). However, at the maximum, the amount of pCREB synthesized in response to forskolin was higher than in the presence of EdTx, CT, and CyaA. The total cellular amount of CREB, which was assayed on the same samples by western blotting with an anti-CREB antibody, did not vary during the treatment with EdTx, CT, CyaA, and forskolin (not shown). These cAMP-elevating agents also induced phosphorylation of the CREB-related transcription factor ATF-1, which was observable by western blot with the anti-pCREB antibody owing to the common presence of a phosphorylated serine epitope (not shown).

### Specificity of CREB phosphorylation by PKA

**Figure 4:**
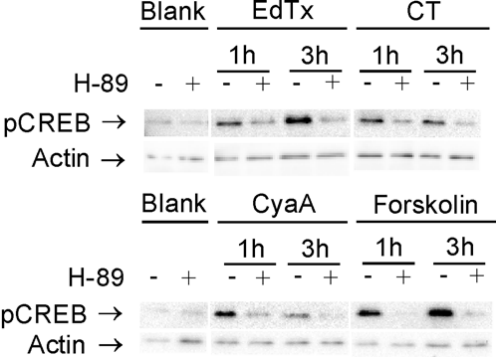
PKA-dependent phosphorylation of CREB upon stimulation of Jurkat cells with EdTx, CT, CyaA, or forskolin. Cells were stimulated for the indicated times with EdTx (10 nM EF+40 nM PA), CT (3 nM), CyaA (5 nM), or forskolin (25 µM) in the presence or absence of 20 µM H-89 dihydrochloride, an inhibitor of PKA, lysed and processed for western blotting using an antibody against S133-pCREB. On each lane 10 µg of protein were loaded. Different loading was controlled by staining against actin. In separate experiments, RSK1/2 and MSK1, which mediate CREB phosphorylation during T cell activation, were found not to be activated following T cell stimulation with EdTx, CT, CyaA and forskolin (not shown). The experiment shown is representative of four performed.

We further addressed the question whether phosphorylation of CREB depended only on PKA or also on other factors that could be activated by cAMP in Jurkat T cells. To this end, we monitored EdTx-, CT-, CyaA-, and forskolin-mediated CREB phosphorylation in the presence and absence of the inhibitor H-89 dihydrochloride, which is commonly used to discriminate between the effects of PKA from those of other kinases, as well as of the guanine nucleotide exchange factor Epac. Epac can induce phosphorylation of CREB in a Rap1- and MAPK-dependent way [38], [42], [43] and was found to mediate EdTx-induced inhibition of endothelial cell chemotaxis [44]. In the presence of 20 µM H-89, CREB phosphorylation induced by EdTx (10 nM EF+40 nM PA), CT (3 nM), CyaA (5 nM), or forskolin (25 µM) was strongly inhibited (Fig. 4). However, H-89 has been reported to affect the activity of mitogen/stress activated kinases [45], which can phosphorylate CREB [39]–[41] and, interestingly, CREB phosphorylation during T cell activation is dependent on RSK2 [46], [47] and MSK1 [48]. We therefore verified whether this kinase family might have been activated in T cells by the cAMP-modulating toxins. Jurkat cells were incubated with EdTx, CT, CyaA, or forskolin over a range of 24 h and analyzed by western blotting using antibodies directed against the active, S380-phosphorylated isoforms 1 and 2 of p90 ribosomal S6 kinase (RSK) and against the active, T581-phosphorylated mitogen- and stress-activated protein kinase (MSK) 1. We found that the phosphorylation levels of RSK1/2 and of MSK1 were not affected by any of the toxin or drug added (not shown).

### Expression of a CRE-driven reporter gene

**Figure 5:**
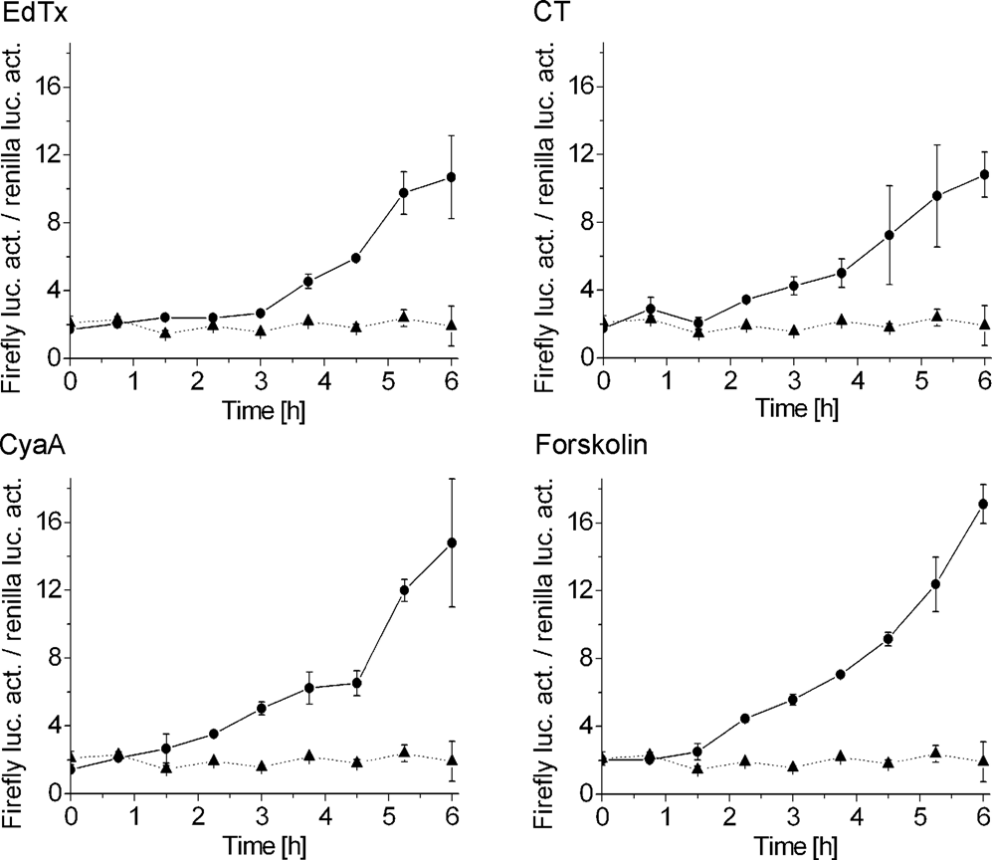
Expression of a reporter gene under the control of CRE upon stimulation of Jurkat cells with EdTx, CT, CyaA, or forskolin. Cells were double-transfected with plasmids coding for firefly luciferase under the control of the inducible promoter CRE and for renilla luciferase under the control of a viral promoter with basal transcriptional activity. Each sample contained 10^5^ cells that were lysed and immediately processed for a chemiluminescence assay. The ratio between the activity of firefly and renilla luciferases is plotted. An increasing value of this ratio indicates that the gene regulated by CRE is expressed. Samples stimulated with EdTx (10 nM EF+40 nM PA), CT (3 nM), CyaA (5 nM), or forskolin (25 µM) are depicted by solid lines with filled circles, whereas untreated samples are represented by dotted lines with filled triangles. EF and PA alone failed to mediate expression of firefly luciferase, whilst DMSO induced a very slight response (not shown). For every sample, firefly luciferase activity per µg of protein in cell lysates was also obtained, giving very similar results (not shown). The data are the mean and standard deviations of four independent experiments performed in triplicates.

To test whether the phosphorylation of CREB described above could lead to gene transcription through its cognate activator sequence cAMP response element (CRE), Jurkat cells were double-transfected with two plasmids, one coding for firefly luciferase under the control of CRE and the other coding for renilla luciferase under the control of a viral promoter with constitutive transcriptional activity. Stimulation of pCREB transcriptional activity in the transfected cells will result in an increase of the ratio of firefly luciferase activity to that of renilla luciferase activity. Indeed, as shown in Fig. 5, EdTx (10 nM EF+40 nM PA), CT (3 nM), CyaA (5 nM), or forskolin (25 µM) were able to stimulate expression of the CRE-controlled gene. In the presence of EdTx no activation of firefly luciferase expression could be detected before 3 h after addition, compared to 1.5 h in the presence of CT, CyaA and forskolin. In the same samples, firefly luciferase activity per µg of protein in cell lysates was also determined and very similar results were obtained (not shown).

### Duration of pCREB binding to chromatin and transcriptional activation

**Figure 6:**
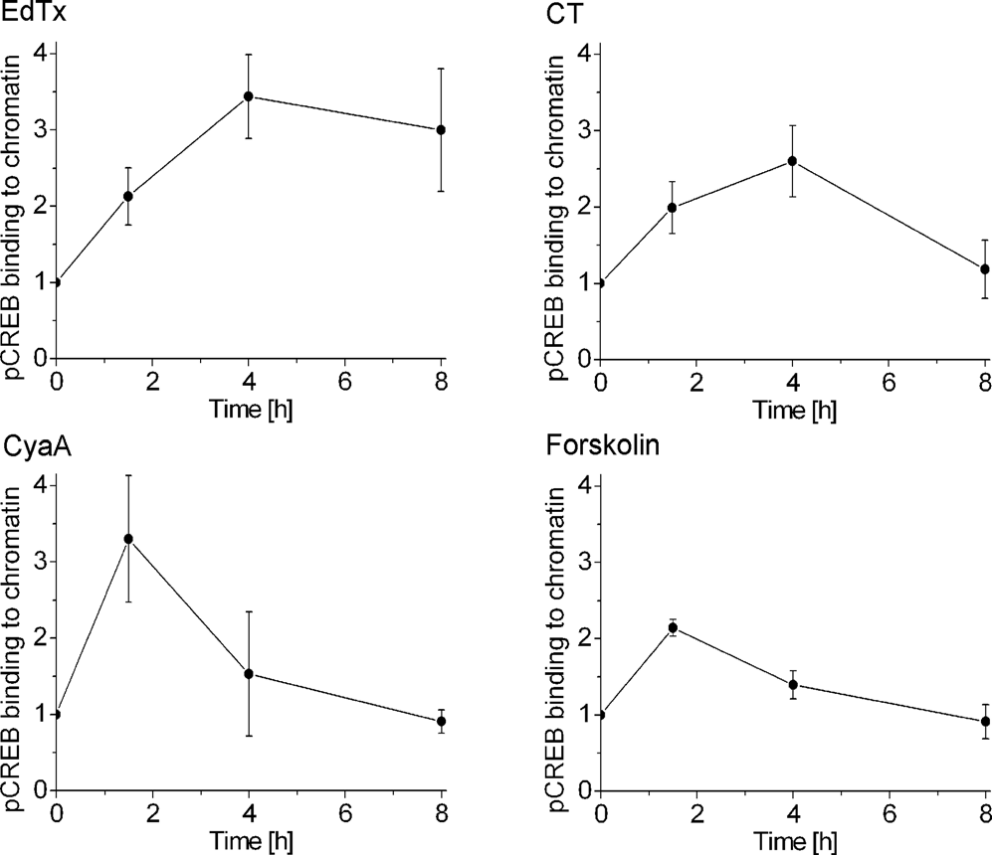
Duration of pCREB binding to chromatin upon stimulation of Jurkat cells with EdTx, CT, CyaA, or forskolin. Cells were treated for the indicated times with EdTx (10 nM EF+40 nM PA), CT (3 nM), CyaA (5 nM) and forskolin (25 µM). Protein and DNA were cross-linked with formaldehyde, then cells were lysed and chromatin was sheared to 600 bp fragments by sonication. Fragmented, transcriptionally active chromatin binding pCREB was recovered by immunoprecipitation of chromatin with a S133-pCREB specific antibody and quantified by western blotting using an antibody specific for acetylated histone H4, which is present in transcriptionally active DNA. The amount of precipitated chromatin at t = 0 was set equal to 1. DMSO, the vehicle of forskolin, induced a very slight increase in pCREB association with transcriptionally active chromatin (not shown). The data shown are the mean and standard deviations of three independent experiments.

The use of a reporter system has shown that EdTx, CT, CyaA, and forskolin are capable of inducing expression of a gene regulated by CRE. However, a CRE-driven reporter assay is a highly simplified system with respect to transcription of cellular genes, where several distinct promoter sequences control this process. Therefore, to test for the association of pCREB with transcriptionally active genomic DNA, we immunoprecipitated pCREB and determined the amount of associated chromatin by western blotting with an antibody specific for the acetylated histone H4, which is present in CREB-activated genes [49], [50]. Fig. 6 shows that EdTx, CT, CyaA, and forskolin are able to promote the association of pCREB with transcriptionally active chromatin, as the amount of precipitated pCREB-bound DNA increased following their addition with respect to untreated samples. However, the duration of pCREB binding to chromatin induced by the different cAMP-elevating agents varied significantly. In fact, whereas EdTx and CT triggered an enduring binding of pCREB to chromatin, the effects of CyaA and forskolin declined much more rapidly.

### Inhibition of CREB phosphorylation in cells pre-treated with cAMP-rising agents

**Figure 7:**
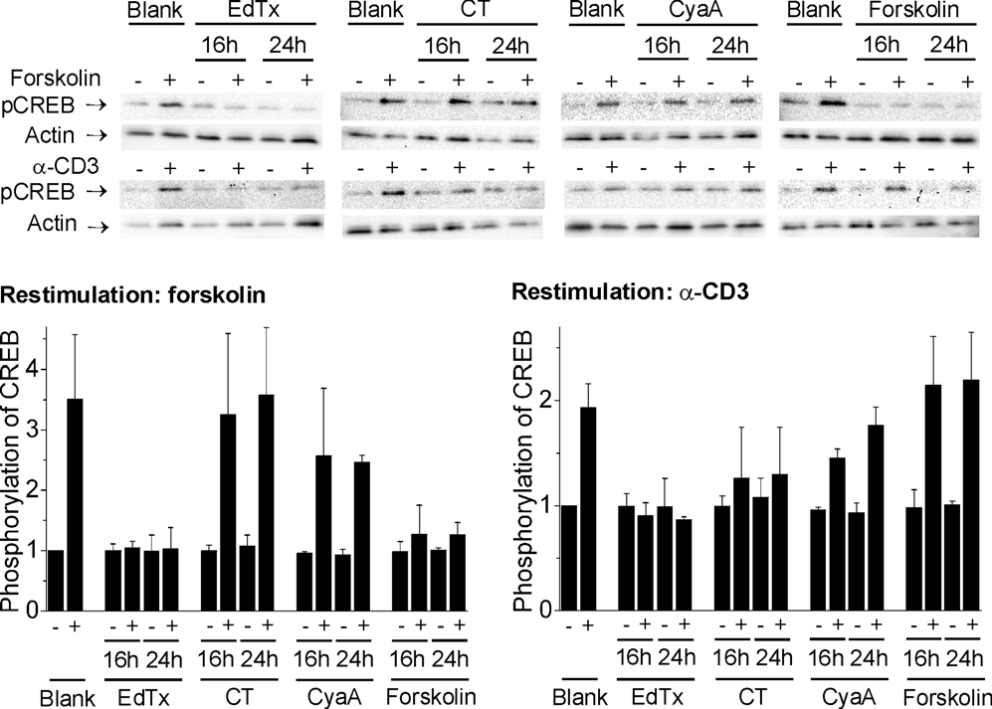
Inhibition of CREB re-phosphorylation in cells pre-treated with EdTx, CT, CyaA or forskolin. Jurkat cells were incubated with EdTx (10 nM EF+40 nM PA), CT (3 nM), CyaA (5 nM), or forskolin (25 µM) and 16 or 24 h after this first treatment, CREB phosphorylation was re-stimulated by addition of forskolin (25 µM) or by α-CD3 antibody mediated cross-linking of the TCR for 30 min. Cells were then lysed and processed for western blotting with antibody against S133-pCREB. The intensity of each band was determined using the software Quantity One (Bio-Rad) and was corrected for different loadings using the corresponding value of actin as control. The value of untreated samples was set equal to 1. The blots show one representative experiment and the graphs give the mean and standard deviation of three independent experiments.

The fact that phosphorylation of CREB caused by EdTx was terminated after 14 h (Fig. 3) in spite of the continuously raising intracellular cAMP concentration (Fig. 1) suggested that negative feedback mechanisms are activated. To test whether these mechanisms could suppress CREB re-phosphorylation after a first activation provoked by EdTx (10 nM EF+40 nM PA), CT (3 nM), CyaA (5 nM), or forskolin (25 µM), we treated Jurkat cells with these cAMP-elevating agents for 16 h or 24 h (at these time points the levels of pCREB had returned to basal) and then re-stimulated CREB phosphorylation by addition of 25 µM forskolin or by anti-CD3 mediated cross-linking of the TCR. As shown in Fig. 7, EdTx abolished re-phosphorylation of CREB induced both by forskolin and by TCR-cross-linking. At variance, CT strongly inhibited TCR-mediated CREB re-phosphorylation, but not CREB re-activation provoked by forskolin. CyaA slightly diminished CREB re-phosphorylation mediated by forskolin and TCR cross-linking. Restimulation of forskolin-treated cells with the same agent, i. e. forskolin, resulted in strong inhibition of CREB re-activation, but allowed CREB re-phosphorylation following TCR cross-linking.

## Discussion

EdTx-mediated suppression of T cell activity is generally thought to be achieved by inhibition of MAPK-dependent signaling. The present data indicate that EdTx also targets CREB-dependent T cell signaling. In fact, cAMP synthesis and phosphorylation of CREB are tightly regulated in space and time during T cell activation [46], [51], [52] and many of the activated genes are regulated by CRE [46]. Therefore, toxin-mediated alterations of the spatio-temporal synthesis of cAMP appears to be a potent way exploited by pathogens to block T cell activity.

We report here that EdTx mediates an extremely prolonged rise of cAMP levels, whereas CT-, CyaA- and forskolin-induced elevations of cAMP concentration are transient. This sustained action might be explained either by enduring catalytic activity of EdTx in the cytosol due to high resistance to proteolytic degradation or be linked to its mode of cell entry. In fact, EdTx exploits the endocytic route to reach the cytosol and was proposed to associate with ILVs [13]. These vesicles may function as toxin stores that, upon gradual back-fusion with the limiting membrane of LEs, could progressively release EdTx into the cytosol to produce high amounts of cAMP over a long period of time [15].

The prolonged rise in cAMP levels elicited by EdTx also resulted in long-lasting phosphorylation of CREB. EdTx, CT, CyaA and forskolin were all able to induce transcription of a reporter gene. However, EdTx and, to a lesser extent, CT appeared to be more efficient in promoting enduring pCREB association with transcriptionally active chromatin. This is consistent with the generally accepted finding that stimuli can provoke phosphorylation of CREB with comparable kinetics and stoichiometry, yet not all stimuli are able to induce pCREB-mediated transcription to the same extent [39], [41]. Clearly, the ability of EdTx, CT, CyaA and forskolin to induce host gene transcription did not correlate with the amount of produced cAMP, as CT mediated only a limited rise of global cAMP levels compared to that triggered by EdTx or forskolin. To the contrary, this effect seems to be strongly influenced by the site of cAMP production. In fact, while CyaA- and forskolin-elicited increase of cAMP concentration is particularly strong below the plasma membrane [16], EdTx [16] and CT [53] cause extensive cAMP rise in the perinuclear region. Indeed, compartmentalization is of prominent importance in cAMP-dependent signaling in many cell types, including T lymphocytes, as it allows specific interactions of PKA with its targets and therefore distinct signaling outputs [54]–[56]. The present data indicate that perinuclear production of cAMP is particularly efficient in inducing pCREB association with transcriptionally active chromatin. Therefore, localization of the enzymatic activity of EdTx to the perinuclear region appears to be a specific feature of its intoxication mechanism with defined functional consequences. A selected cytosolic localization of the adenylate cyclase activity could explain the reports that *Pseudomonas aeruginosa* ExoY has distinct effects on endothelial cell permeability with respect to the cAMP produced at the level of the plasma membrane [57]–[59].

CREB mediates expression of a high number of genes that are implicated in a wide array of cell functions, including transcription, cell cycle, and immune regulation [41], [50]. We found that EdTx-elicited CREB phosphorylation is specifically mediated by PKA, whereas, during T cell activation, CREB phosphorylation induced by cross-linking of TCR is mainly mediated by proteins of the MAPK pathway, in particular RSK2 [46], [47] and MSK1 [48]. RSK1/2 and MSK1 were not phosphorylated upon stimulation with EdTx, in keeping with the finding that EdTx blocks phosphorylation of their upstream kinases MEK and ERK [22], [24]. This suggests that EdTx and TCR cross-linking may elicit distinct genetic programs. For instance, the genes coding for IL-2, IL-2 receptor, interferon-γ, tumor necrosis factor α, and the early activation marker CD69 contain CRE sites [41], [46], [60], but their expression was inhibited by EdTx [24]. On the contrary, PKA was reported to downregulate the expression of cyclin D3 and up regulate p27^kip1^, which block cell growth [61], [62]. This mechanism could also partially account for EdTx-induced suppression of T cell proliferation [22], [24].

We also report here that, although EdTx initially induces CREB phosphorylation and gene expression, after a prolonged incubation it clearly inhibits the subsequent phosphorylation of CREB induced by forskolin or by TCR cross-linking. This property likely depends on the massive and long-lasting cAMP synthesis caused by EdTx, which probably fuels negative feedback mechanisms of CREB phosphorylation [41]. Interestingly, elevated cAMP levels and hyperactivation of PKA have been implicated in T cell anergy and dysfunction [63]. Possibly, the site of cAMP production might also contribute to inhibition of CREB phosphorylation. Indeed, CT, which similarly induces cAMP elevation in the perinuclear region, was also able to suppress CREB phosphorylation provoked by TCR cross-linking, whereas CyaA and forskolin had a limited effect. These findings indicate that intoxication with EdTx prior to bacterial stimulation of T cells may suppress their activity by preventing an essential step in T cell activation, that is CREB phosphorylation, and thereby allow the pathogen to escape the immune response. Further, a two-phase mechanism of action could contribute to explain the *in vivo* observation that the effects on T lymphocyte cytokine secretion change with the duration of EdTx intoxication [22]. This subversion mechanism of host cell functions, by untimely phosphorylation of CREB followed by inhibition of CREB activity, is likely to operate in other cell types besides T cells, as EdTx-mediated CREB phosphorylation was observed also in macrophages [64]–[66]. Moreover, this mechanism of action may be relevant to a number of bacterial toxins hitting upon host cell signaling, as suggested by the fact that not only EdTx but also CT is able to impair TCR-mediated CREB phosphorylation.

In conclusion, we identify PKA and CREB as targets of inhibition of T cell function caused by EdTx. Our data suggest that EdTx displays its action in two distinct phases: first, EdTx induces PKA-dependent CREB phosphorylation and expression of genes under the control of CRE; then, EdTx prevents CREB phosphorylation in response to incoming stimuli. This is a novel mode of intoxication by bacterial toxins acting on cellular signaling. Comparison of EdTx activity with that of other cAMP-elevating agents such as CT, CyaA and forskolin showed that EdTx is particularly efficient in affecting T cell function, probably owing to the duration and site of cAMP production.
