# Correction: Anthrax Edema Toxin Modulates PKA- and CREB-Dependent Signaling in Two Phases

**DOI:** 10.1371/annotation/b72b80a6-ee81-49e6-a086-0292f6255d4f

**Published:** 2008-11-05

**Authors:** Andrea Puhar, Federica Dal Molin, Stéphanie Horvath, Daniel Ladant, Cesare Montecucco

The fourth author's name was spelled incorrectly. It should read: Daniel Ladant. This also affects the article's citation. The corrected citation should read: Puhar A, Dal Molin F, Horvath S, Ladant D, Montecucco C (2008) Anthrax Edema Toxin Modulates PKA- and CREB-Dependent Signaling in Two Phases. PLoS ONE 3(10): e3564. doi:10.1371/journal.pone.0003564

